# Transitioning from Regular Electrolytes to Solvate Ionic Liquids to High-Concentration Electrolytes: Changes in Transport Properties and Ionic Speciation

**DOI:** 10.1021/acs.jpcc.4c02248

**Published:** 2024-07-10

**Authors:** Ernest O. Nachaki, Daniel G. Kuroda

**Affiliations:** Department of Chemistry, Louisiana State University, Baton Rouge, Louisiana 70803, United States

## Abstract

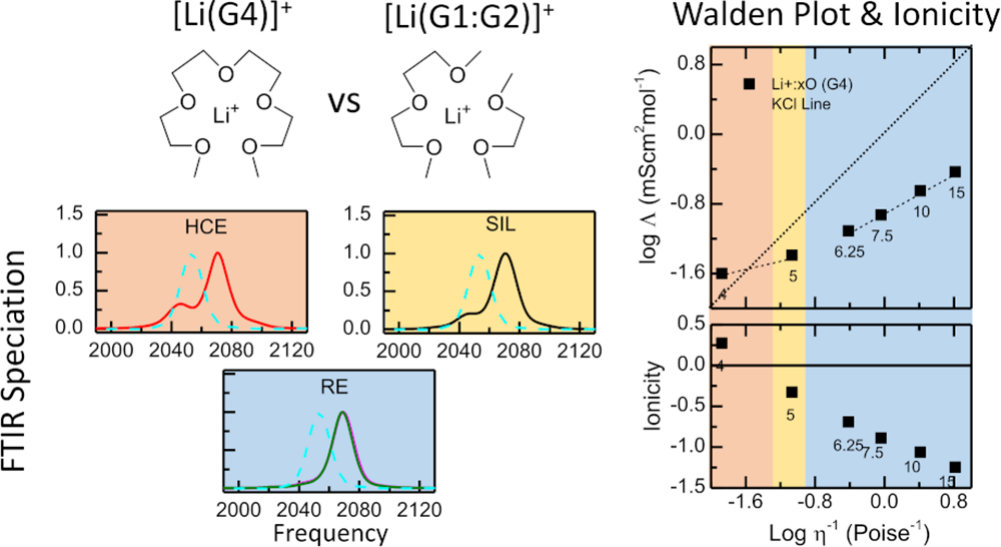

Glyme-based lithium-ion electrolytes have received considerable attention from the scientific community due to their improved safety, as well as electrochemical and thermal stability over carbonate-based electrolytes. However, these electrolytes suffer from major drawbacks such as high viscosities. To overcome the challenges that hinder their full potential, the molecular description of glyme-based lithium electrolytes in the high-concentration regime, particularly in the solvate ionic liquid (SIL) and high-concentration electrolyte (HCE) regimes, is needed. In this study, model glyme-based electrolytes based on a lithium thiocyanate and either tetraglyme (G4) or a mixture of monoglyme (G1) and diglyme (G2) were investigated as a function of the solvent-to-lithium ratio using linear and nonlinear IR spectroscopies, in combination with ab initio computations as well as electrochemical methods . The transport properties reveal enhanced ionicities in the HCE and SIL regimes ([O]/[Li] ≤ 5) compared to the regular electrolytes (REs, with [O]/[Li] > 5) in both pure (G4) and mixed (G1:G2) glymes. IR and ab initio computations relate these larger ionicities to the higher concentration of charged aggregates in the HCE and SIL electrolytes ([O]/[Li] ≤ 5). Moreover, it was observed that the use of mixed glymes appears to have a minimal effect on the transport properties of REs but exhibits deleterious effects on SILs. Overall, the results provide a molecular framework for describing the local structure of lithium glyme-based electrolytes and demonstrate the key role that the nature of glyme solvation plays in the molecular structure and consequently the macroscopic properties of the Li-glyme SILs, HCEs, and REs.

## Introduction

Current commercial Li-ion batteries (LIBs) utilize liquid electrolytes composed of a lithium salt and a mixture of organic carbonate solvents.^1^ The standard LIB electrolyte consists of a 1 M solution of lithium hexafluorophosphate (LiPF_6_) in a mixture of ethylene carbonate (EC) and linear carbonate, such as dimethyl carbonate (DMC).^2^ This electrolyte confers balanced electrochemical properties, such as the inhibition of aluminum corrosion,^3,4^ a relatively high ionic conductivity,^5^ and the formation of a stable solid electrolyte interphase (SEI).^6,7^ However, this standard electrolyte also suffers from the instability of the LiPF_6_ salt at high temperatures, the high viscosity of EC at low temperatures that hinders rate capabilities,^8,9^ and the unsuitability for high voltages due to electrochemical reactivity under these conditions.^10^ Attempts to replace the LiPF_6_ with other stable salts, such as lithium bis(trifluoromethanesulfonyl)imide (LiTFSI), have resulted in problems related to aluminum dissolution and the formation of unstable SEI.^8^ The latter is particularly important because it typically results in dendrite growth and consequently a short circuit event or combustion from thermal runaway.^11^ While high-voltage cathode materials have been developed, the electrolyte problems described above underscore the need for safer options for high-voltage applications.

Strategies to circumvent the electrolyte challenges have included the use of high concentrations of the lithium salt in the organic solvents.^12−15^ These high-concentration electrolytes (HCEs) have been shown not only to improve the electrochemical stability but also to mitigate the dissolution of aluminum current collectors.^12,16,17^ In addition, HCEs are electrochemically stable because they form a stable SEI in the absence of EC and LiPF_6_,^15,18^ which suppresses dendrite formation.^15,19^ The HCE is also inherently safer due to its reduced solvent content.^18,20,21^

The typical commercial LIB electrolyte^22−24^ is fundamentally regarded as a high-concentration solution because its ionic species interact with each other and hence do not fulfill the Debye–Huckel model of a dilute solution.^25^ However, the term HCE has been used for electrolytes with salt concentrations well beyond the typical 1–1.5 M.^5,12,23,24,26−31^ Previous studies have defined HCEs differently. One of the earliest studies of HCEs using LiTFSI in acetonitrile at a 1:1.9 salt:solvent molar ratio (>4.2 M) referred to it as a superconcentrated electrolyte (SCE).^12^ Other studies involving electrolytes having a salt:solvent molar ratio of 1:1.9 or higher termed their electrolytes as HCEs.^27,32,33^ Regardless of their definition, these studies demonstrated that SCEs or HCEs have a number of solvent molecules that do not fulfill the lithium-ion (Li^+^) tetrahedral coordination structure of [Li(solvent)_4_]^+^, for a monodentate solvent. Under such conditions, the counterion participates in the Li^+^ coordination, leading to the formation of ion pairs, aggregates,^20,27,34^ and ionic networks.^35−37^ The distinct Li^+^ solvation structures in HCEs are responsible for the atypical electrolyte properties, such as the enhanced oxidative stability,^23,34^ thermal stability,^38^ and improved Li^+^ transport.^34,39,40^

Solvate ionic liquids (SILs) have been shown to have similar properties to HCEs and ionic liquids (IL).^41−44^ These properties include thermal stability,^45^ a wide electrochemical stability window, and the ability to withstand high anodic voltages of up to 5 V.^34^ Moreover, previous studies have also shown that SILs are able to inhibit the corrosion of Al current collectors.^46,47^ This makes SILs promising candidates for high-voltage battery applications,^48^ with the advantage of eliminating the competition between similarly charged ionic species, such as that observed in a solution of a lithium salt in an IL.^49^ A SIL is typically described as a solvated cation and an anion. The solvated ionic species is created by a solvent (i.e., glyme) that chelates the cation by fulfilling the 4–5 coordination sites of Li^+^ with a single solvent molecule.^48,50^ One factor that influences the formation of a SIL is the Li^+^-glyme interaction as a greater interaction ensures the stability of the solvated cation.^51^

Henderson had previously ranked the ionic association strength of common solvated anions to Li^+^ in the following order (from stronger to weaker): CF_3_CO_2_^–^ > NO_3_^–^ > Br^–^ > CF_3_SO_3_^–^ > BF_4_^–^ > SCN^–^ > I^–^, ClO_4_^–^ > PF_6_^–^ > AsF_6_^–^, SbF_6_^–^, TFSI^–^, BETI^–^ > BPh_4_^–^.^52^ Watanabe and co-workers have also found a similar ranking (TFSI^–^ > ClO_4_^–^ > BF_4_^–^ > OTf^–^ > NO_3_^–^> TFA^–^.), but using the stability of the [Li(glyme)]^+^ complex.^50^ Hence, “good” SILs have been regarded as combinations of 1:1 molar ratios of a lithium salt containing either TFSI^–^ or ClO_4_^–^ and trigylme (G3) or tetragylme (G4) because they form very stable solvate cations, [Li(G3)]^+^ or [Li(G4)]^+^, respectively. However, it has also been observed that some combinations of lithium salts and glyme mixtures exhibit properties that slightly deviate from those of good SILs, but cannot be simply regarded as regular electrolytes (REs) and have therefore been referred to as “poor” SILs. Such systems include a lithium salt in monoglyme (G1) and diglyme (G2), such as [Li(G1)_*n*≤3_]TFSI, [Li(G2)_*n*≤3_]TFSI, and lithium salts with either trigylme or tetragylme, such as [Li(G3)]^+^ or [Li(G4)]^+^ with any of the following anions: NO_3_^–^, OTf^–^, and BF_4_^–^.^48,50,53,54^ While SILs present excellent properties for their practical application in LIBs, their implementation has not yet been realized due to a number of challenges, such as low lithium transport numbers^14^ and the presence of free glymes that could affect the long-term electrochemical stability.^48^

Previous attempts to improve ion transport in HCEs and SILs have focused on the use of diluents to reduce viscosities and improve conductivities.^14^ However, the diluted HCEs still suffer from competition between the diluent and the glyme for solvating the Li^+^, which affects the stability of the solvated cation.^55^ In addition, this approach often results in electrolytes with low electrochemical stability from the diluents, and reduced ionicities from the formation of ion pairs.^56^ Another approach relied on mixtures of glymes with nonstoichiometric Li–glyme ratios to produce stable solvate complexes.^46^ In particular, the [Li(G2)_4/3_][TFSI] solution has a self-diffusion coefficient ratio of *D*_Glyme_/*D*_Li_ = 1.02 and thermal stability of up to 130 °C,^46^ but its viscosity is still considerably high, resulting in low ionic conductivity. A previous computational study utilizing the Onsager transport coefficients to understand the ionic motion contributions to the total ionic conductivity (σ_ion_) showed that the observed low conductivities in a SIL with a long-chain glyme (LiTFSI:G4) are caused by the strong anticorrelated ionic motions that are suppressed in its short-glyme analogue.^57^ In addition, the change in solvation structure increases the transfer number due to a decrease in the stability of the [Li(G1/2)]^+^ complex when compared to [Li(G4)]^+^. This observation, which was later experimentally validated,^58^ emphasizes the need to study the effect of the SIL local structures and glyme-based HCEs. In another study of G3 and G4 SILs, an enhancement in the transfer numbers was observed for anions with high Lewis basicity (i.e., TFSI^–^ < ClO_4_^–^ < BF_4_^–^ < OTf^–^ < NO_3_^–^ < TFA^–^),^59^ but their ionicities and ionic conductivities decreased.^5^ Thus, it appears that there is a trade-off between transfer number and ionic conductivity and that a balanced Li^+^ transport would be achieved with anions of moderate Lewis basicity, such as BF4^−^, OTf^−^ and likely SCN^−^,^52^ further justifying the investigation of such systems. Finally, attempts have been made to improve the SILs properties by adding an excess of the lithium salt to convert them to HCEs (i.e., [O]/[Li] < 4).^39^ These HCE-SIL electrolytes have been shown to have improved oxidative stability and Li^+^ transport but low ionic conductivity due to their high viscosities.^38,60^ These studies demonstrate that to enhance the ionic transport of SILs and HCE-SILs while maintaining their advantageous electrochemical properties, it is critical to have a molecular understanding of the influence of ion–solvent and ion–ion interactions on their transport properties.

Many experimental investigations of the Li^+^ solvation environments in HCEs and SILs have been pursued, but their molecular structures and their relationship to their transport properties have remained elusive. For example, a previous study using X-ray diffraction and Raman spectroscopy investigated the molecular structures and mechanism contributing to the faster Li^+^ transport in sulfolane (SL)-based HCEs.^40^ However, the overlap of the Raman spectral signatures rendered an incomplete picture of the Li^+^ solvation environment.^40^ Another study of LiTFSI in G3/G4 SIL (1:1) and HCE-SIL (LiTFSI/glyme >1) utilized the vibrational modes of the TFSI anion, to infer the strength of Li–glyme interaction and the speciation of the system: contact ion pairs (CIPs) and aggregates (AGGs).^39^ However, it has been shown that these Raman modes are not well suited for assigning ionic speciation because of an ambiguity in the spectroscopic signatures for the different ionic species.^61^ Other studies have used indirect methods, such as the NMR self-diffusion coefficient ratios and thermal stability of HCEs and SILs, to deduce the possible local structure of Li^+^.^38,62^ Molecular dynamics simulations have also been applied to explore the Li^+^ solvation structure and dynamics in HCEs, but most studies do not have the accuracy to reproduce experimental observations.^38^ Overall, these previous studies reveal an incomplete picture of the Li^+^ solvation environment in HCEs and SILs, highlighting the need for a better description of the Li^+^ solvation structure and dynamics in relation to the transport properties.

**Scheme 1:**
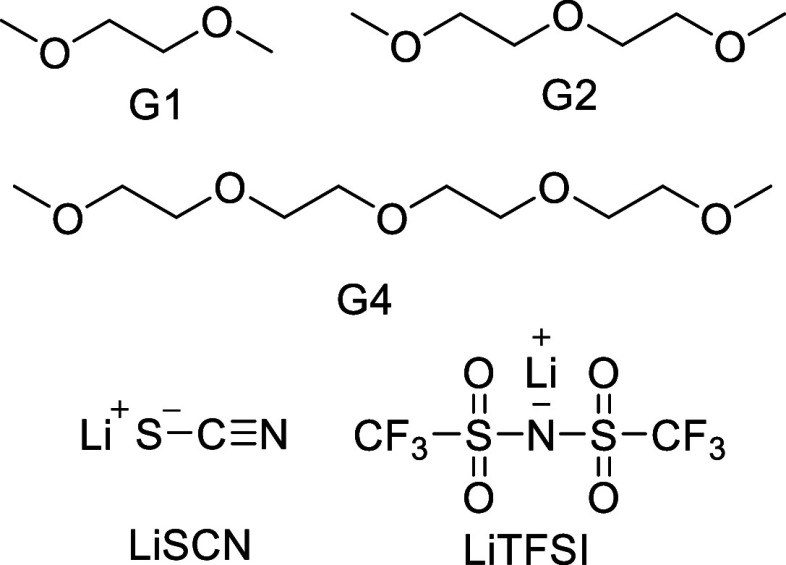
Structures of Glymes: Monoglyme (G1), Diglyme (G2), and Tetraglyme (G4), and Li Salts: Lithium Thiocyanate (LiSCN) and Lithium *Bis*(trifluoromethanesulfonyl)amide (LiTFSI)

Infrared spectroscopy is a powerful tool for probing solvation structures, but the direct investigation of these highly concentrated electrolytes has remained elusive due to the lack of intrinsic IR probes (i.e., well-localized vibrational modes) that can report the local Li^+^ environment. For example, neither the glyme molecules (from G1 to G4) nor the anions (TFSI^–^, ClO_4_^–^, BF_4_^–^, OTf^–^, TFA^–^) typically found in the Li-glyme HCEs and/or SILs possess such well-localized IR modes.^50^ In addition, the very high salt concentrations complicate the experimental characterization of these systems. In this study, the local structures related to the transport properties of Li^+^-glyme HCEs and SILs are investigated by using LiSCN in glymes at different concentrations. In addition, this study explores the effect of pure versus mixed glyme solvation on the molecular structure and transport properties. To this end, two solvents were investigated (Scheme 1): one containing pure G4, which is capable of fully coordinating the Li^+^ with a single solvent molecule and the other consisting of a mixture of G1 and G2 at a molar ratio of 1:1, which is equivalent to G4 in terms of the number of oxygen atoms. It should be noted that the solutions containing mixed glymes systems cannot be regarded as SILs, and they are only used to elucidate the effect of mixed glyme solvation on such SIL-like systems. In particular, the G1:G2 mixture and the G4 molecule have the same number of oxygen atoms allowing us to separate the solvent cooperativity effect observed in the G4 glyme. The choice of the LiSCN salt is based on the presence of the CN stretch, which is a well-localized intrinsic IR probe and sensitive to the chemical speciation.^63−67^ However, the choice of this anion is not purely conceptual. Based on the Li^+^-anion interactions,^52^ the [Li(G4)]SCN should form a SIL, with similar properties to those of LiBF_4_ in G4, as shown by the Watanabe group.^51^ In addition, there has been a proof of concept for the possible use of SCN^–^-based electrolytes for Li–S batteries.^68^ Finally, the LiSCN also forms HCEs, where the number of available oxygen atoms from the glyme molecule is less than the 4 required to fully solvate Li^+^.

Here, the effect of the solvation and ionic speciation of the LiSCN-glyme on the transport properties is determined as a function of the Li^+^–glyme ratio (i.e., RE, SIL, and HCE regimes) and the nature of glyme solvation. For consistency, the LiSCN-glyme mixtures with [O]/[Li] > 5 are defined as regular electrolytes (REs), with [O]/[Li] = 5 as SILs and [O]/[Li] < 5 as HCEs, for both pure and mixed glymes. Note that the REs and HCE classification simply implies an excess of solvent or salt, respectively. While the transport properties of these electrolytes were determined from densities, viscosities, and ionic conductivities; the atomic-level interactions and the Li^+^ solvation environments as a function of the glyme identity were obtained using IR spectroscopies (FTIR and 2DIR) in combination with ab initio calculations (DFT).^69−72^ In particular, 2DIR spectroscopy is used to reveal the molecular relationship between different solvation structures.^69,73,74^

## Methods

### Sample Preparation

Lithium bis(trifluoromethanesulfonyl)amide (LiTFSI, 99% Oakwood Chemicals) was dried in a vacuum oven at a temperature of 150 °C for a duration of 48 h. Initially, lithium thiocyanate hydrate (LiSCN·*x*H_2_O, LiSCN > 63% Thermo Scientific) was dried at a temperature of 70 °C for 24 h in a vacuum oven, followed by drying at 110 °C for an additional 48 h. Tetrabutylammonium thiocyanate (TBASCN, 95% TCI Chemicals) was used as received. Monoglyme (2-dimethoxyethane ≥99.5% Millipore Sigma), diglyme (bis(2-methoxyethyl) ether ≥99.0% Acros Organics), and tetraglyme (tetraethylene glycol dimethyl ether >99.0% Acros Organics) were dried in 4 Å molecular sieves for at least 48 h before use. For the pure glyme electrolyte, LiSCN was dissolved in G4 in ratios of 1:0.8 (HCE), 1:1 (SIL), and 1:1.5, 1:2, 1:3 (REs), while for the mixed glyme electrolyte solutions of LiSCN in 1:1 mixture of G1 and G2, LiSCN was prepared in ratios of 1:0.5, 1:0.625, 1:0.75 (HCEs), 1:1 (SILs), and 1:2, 1:3 (REs). A table of conversion between molar ratios to [O]/[Li] to concentration is found in the Supporting Information. Note that the HCEs of G4 at concentrations higher than 1:0.8 could not be measured because they were not liquids. The SIL and HCEs electrolytes were prepared by a series of gentle heating of the salt-solvent mixture to ≈40 °C and vortexing cycles, repeatedly for at least 2 h. All samples were prepared in a nitrogen-filled glovebox.

### Rheology and Ionic Conductivity Measurements

The viscosity measurements of the solutions were performed at 25 °C using the Brookfield DVI-II+ Pro. Ionic conductivity measurements were performed using a WaveDriver 100 Potentiostat (PineResearch). The ionic conductivities of the electrolyte solutions were measured using the potentiostatic electrochemical impedance spectroscopy (EIS-POT) technique, using a ceramic platinum screen printed electrode (Pt-SPE, RRPE2011PT-6 PineResearch), consisting of 2 mm platinum working electrode (WE), a platinum counter electrode (CE), and a silver pseudoreference electrode. For all EIS measurements, the electrochemical cell was allowed to equilibrate for at least 45 min to stabilize the potential of the pseudoreference electrode. The electrochemical cell constant for the ionic conductivity vs EIS-measured cell resistance was determined using potassium chloride (KCl) solutions. All EIS measurements of the Li-glyme electrolytes were performed in a nitrogen-filled glovebox with positive pressure at 25 °C.

### Linear IR Spectroscopy

Fourier transform infrared (FTIR) measurements were conducted using a Bruker Tensor 27 equipped with a liquid nitrogen-cooled MCT detector with a resolution of 0.5 cm^–1^. A Pharmacia Biotech circulating bath was used for temperature control, with a temperature regulation of ±0.1 °C, in conjunction with a Harrick temperature-regulated sample cell comprising 2 mm CaF_2_ windows. Every FTIR spectrum was the result of an average of 40 scans. All FTIR measurements were done with less than 5 μL of sample between two CaF_2_ windows with no spacer and compressed to keep the optical density of the CN stretch of SCN^–^ within the detector linear range.

### Nonlinear IR Spectroscopy

Experiments involving two-dimensional infrared spectroscopy (2D IR) were conducted using a setup analogous to what is detailed in the literature.^75^ In brief, a broadband IR pulse, approximately 60 fs in temporal width, was produced using a Spectra-Physics Spitfire Ace Ti:sapphire amplifier at a 5 kHz repetition rate. This was coupled with an optical parametric amplifier (Spectra-Physics OPA-800C) and an AgGaS_2_ difference frequency generation crystal. Every IR pulse was divided into three identical pulses with wavevectors *k*1, *k*2, and *k*3. These were then focused onto the sample using a lens in a boxcar arrangement. A photon echo is generated in the phase-matching direction of −*k*1 + *k*2 + *k*3. This echo is then combined with a fourth pulse, the local oscillator (LO), spread out by a Triax Horiba monochromator with 100 grooves/mm, and captured by a nitrogen-cooled MCT array detector from Infrared Systems Development consisting of 64 elements. The time intervals between the pulses, represented as τ (between pulse 1 and 2), *T*_w_ (between pulse 2 and 3), and *t* (between pulse 3 and the photon echo), were adjusted using computer-controlled translational stages from PI Micos. The waiting time (*T*_w_) was scanned from 0 to 50 ps, with an exponential increase consisting of 21 time-steps; τ time was varied from −3.5 to 3.5 ps, with a time step of 5 fs. The LO consistently came before the signal by approximately 1 ps. A double Fourier transform with respect to times τ and *t* was employed to convert the signal from the time domain (*S* (τ, *T*_w_, *t*)) to the frequency domain (*S* (ωτ, *T*_w_, ω*t*)). The complete information on the Fourier transform analysis can be found in the literature.^76^ The 2DIR spectra of the HCEs and SILs were measured using a sample cell consisting of a convex lens CaF_2_ IR window (with a focal length of 1 m) and a regular window with no spacer as previously reported,^77^ in order to decrease the signal absorbance from the sample.

### Density Functional Theory (DFT) Calculations

Gas-phase DFT calculations were performed using Gaussian 16 software,^78^ PBEPBE level of theory, and 6-31+G** basis set.^79−82^ The starting molecular structures, deduced from experiments and consisting of CIPs, neutral and charged aggregates (AGGs) (see the Supporting Information), were built using the Avogadro software^83^ and minimized using the classical force field MMFF94.^84^ The PBEPBE functional and the 6-31+G** basis set were chosen because they have been previously demonstrated to provide a good balance between computational cost and accuracy in modeling the Li^+^ solvation shells.^85^ Geometry optimization of the initial solvation structures was carried out in the gas phase with explicit Li^+^ first solvation shell. This was done because evidence suggests that introducing a dielectric continuum does not alter the thermodynamic patterns of Li^+^ solvation structures.^86^ After the geometry optimizations, the CN stretch IR frequencies of the SCN^–^ anions in the different ionic speciation environments were calculated, and the absence of any imaginary frequencies indicated that the systems were at an energy minimum.

## Results

### Transport Properties

**Figure 1:**
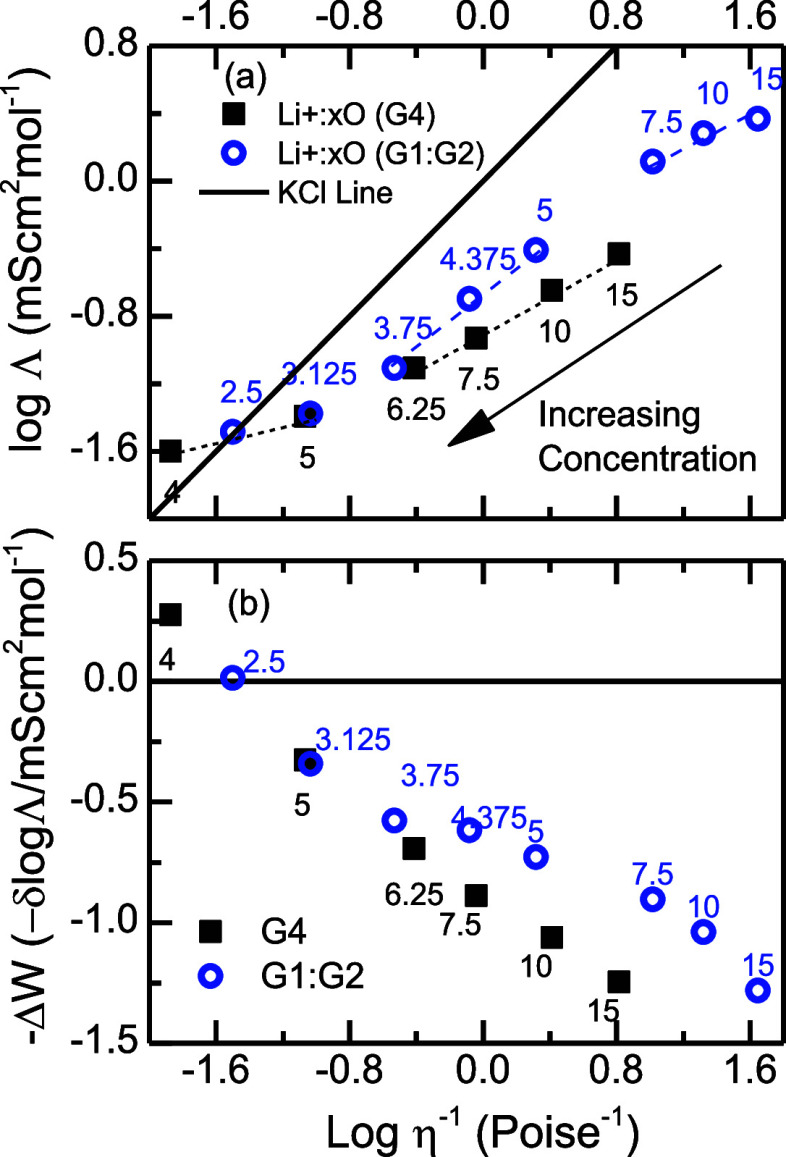
Panel (a) shows the Walden plot of pure glyme systems (black), mixed glyme system (blue), and the ideal KCl line (black); the dashed lines are a guide to the eye. Panel (b) shows the deviations between the measured log Λ_m_ (experimental) and log Λ_KCl_ (“ideal”), as the estimated ionicities (−Δ*W* = −log Λ_m_/Λ_KCl_). The data point labels represent the number of oxygen atoms per Li^+^.

The viscosity (η) and the molar ionic conductivities (Λ_m_) of the G4 and G1:G2 electrolyte solutions were determined as a function of the Li^+^:solvent ratio (either LiSCN[G4]_*x*_ or LiSCN[G1:G2]_*x*_, where *x* = 0.5–3 and thus [O]/[Li] = 5*x*) at 25 °C. The Walden plot of the Li-glyme electrolyte solutions showing the transport properties of the different electrolyte systems is presented in Figure 1. It is observed that both systems evolve toward more ideal electrolytes (defined by the solid line representing KCl_(aq)_) as the Li^+^:solvent molar ratio is decreased. Moreover, it appears that the system undergoes significant changes upon transition from RE to SIL to HCE, as seen by the change in the slope of the Walden plot (WP).

The relative transport properties of the two electrolyte solutions, as seen in the Walden plot, were assessed using the ionicity (Figure 1) as a comparative metric.^5,36,39,87^ Here, the ionicity (−Δ*W*) was estimated from the vertical deviations (Δ*W*) of the conductivity and fluidity points of the Walden plot from the reference line defined by the KCl system.^88−90^ The ionicity shows the following trend HCEs > SILs > REs for both G4 and G1:G2 systems, indicating that HCEs and SILs have higher ionicities than REs. Moreover, REs ([O]/[Li] > 5) formed by either G4 or G1:G2 with the same [O]/[Li] ratio present similar ionicities, but the former has lower fluidity. In contrast, the HCEs and SILs ([O]/[Li] ≤ 5) show higher ionicities for the G4 electrolytes than the G1:G2 analogues, even at the same [O]/[Li] ratio (Figure 1). The changes in the ionicity as the electrolytes shift from the REs to the SILs and later to the HCEs cause the observed change in the WP slope for both G4 and G1:G2 systems (Figure 1). In general, both the G4 and G1:G2 REs present different WP slopes when compared to their corresponding SILs or HCEs. However, the G4 HCEs ([O]/[Li] = 4–5) systems exhibit a slope smaller than that of the G1:G2 SIL system, revealing a smaller change in molar conductivity with viscosity for G4 systems than in the G1:G2 analogue. In the case of the HCEs, the G1:G2 systems appear to have a WP slope similar to that of the G4 HCEs. Overall, it is deduced from the Walden plot and ionicities that the HCEs and SILs exhibit improved transport properties compared to those of the REs, despite their significantly higher viscosities. It is also observed that the G4 HCEs achieve similar transport properties as the G1:G2 HCEs but at lower salt concentrations.

### Solvation Structure

**Figure 2:**
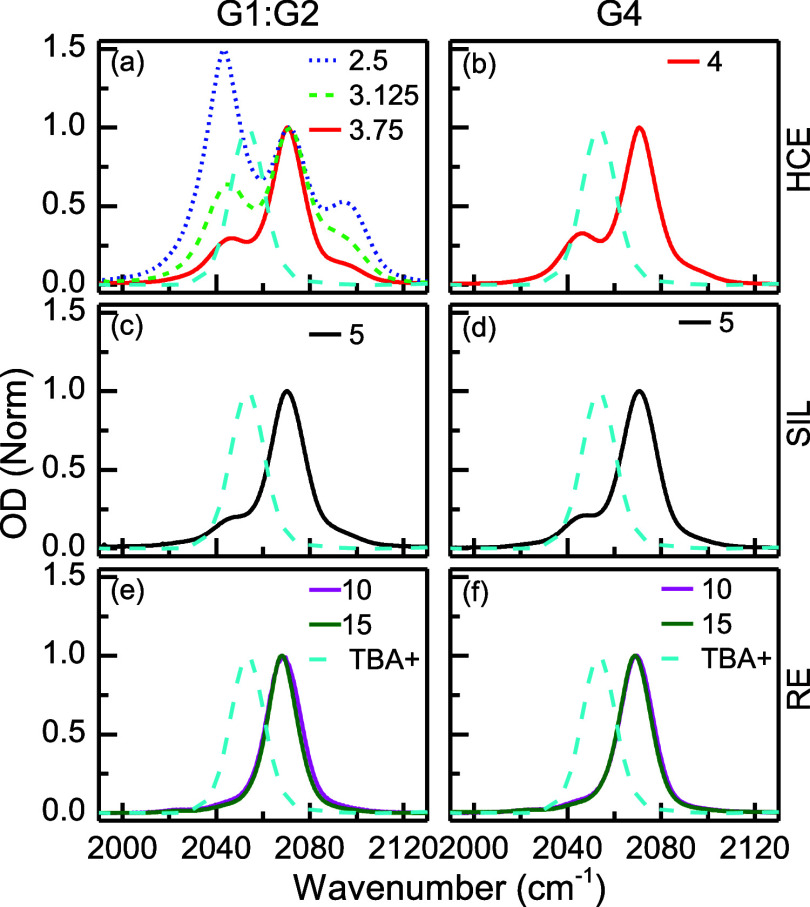
Concentration-dependent FTIR (CD-FTIR) spectra in the CN stretch region for different numbers of oxygen atoms per Li^+^ (legend). Panels (a), (c), and (e) represent LiSCN in G1:G2. Panels (b), (d), and (f) represent LiSCN in G4 at the HCE, SIL, and RE regimes, respectively. The dashed cyan line in panels (a–f) represents TBASCN in G4. All FTIR spectra are normalized with respect to the center peak at ∼2072 cm^–1^.

The molecular structure of the Li-glyme systems was investigated by FTIR spectroscopy using the CN stretch region (∼2000 to 2130 cm^–1^) of the SCN^–^ (Figure 2). The normalized IR spectra of LiSCN in both G4 and G1:G2 (Figure 2) show a major peak at ∼2072 cm^–1^, with two shoulder peaks on either side at ∼2043 and ∼2095 cm^–1^, which grow with increasing Li^+^ concentration. In particular, the shoulder peaks are not present in the RE regimes of G4 and G1:G2 ([O]/[Li] = 10, 15) but appear in their corresponding SIL ([O]/[Li] = 5) and HCE ([O]/[Li] < 5) regimes as shown in Figure 2.

The CN stretch IR signatures in the G1:G2 systems have a direct correspondence to those in the G4 systems (Figure 2), indicating a similar anion speciation in both systems as a function of concentration. However, in the HCE regime (i.e., [O]/[Li] < 5), the pure glyme electrolyte (G4) shows a slightly higher shoulder band than the G1:G2 electrolyte (Figure 2), despite the former having a higher [O]/[Li], or equivalent, a lower concentration salt concentration. To assess the SCN^–^ speciation, a sample of TBASCN in G4 was also investigated. This sample provides a spectral signature of what is considered to be the free anion given that the presence of the bulky tetrabutylammonium cation has minimal effect on the SCN^–^.^91,92^ The CN stretch band of SCN^–^ in the TBASCN G4 sample appears at ∼2053 cm^–1^, which is blueshifted (∼8 cm^–1^) from the lower frequency shoulder peak and redshifted (∼17 cm^–1^) from the central peak. The comparison shows the absence of free SCN^–^ in any of the investigated samples.

**Figure 3:**
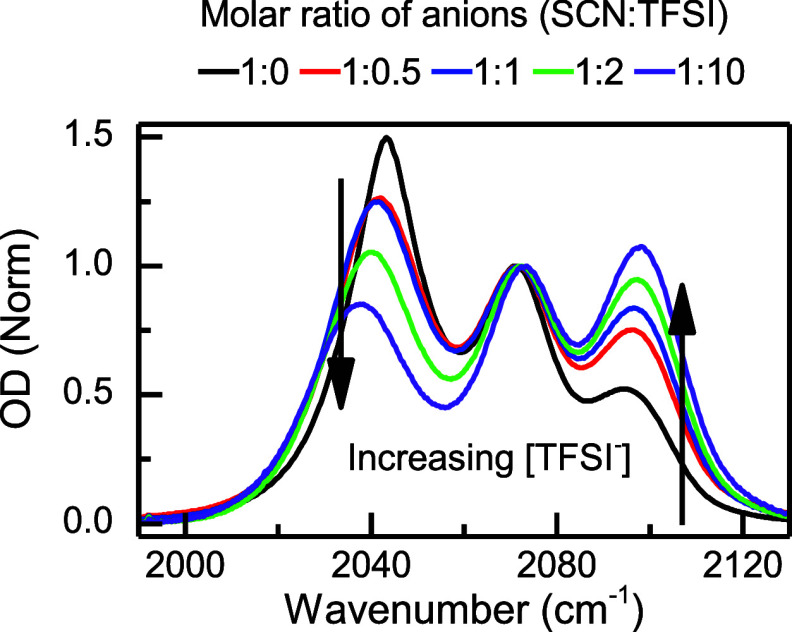
FTIR spectra in the CN stretch region for mixtures of LiSCN and LiTFSI in G1:G2; the black arrows indicate the change in the normalized IR intensity with increasing TFSI^–^:SCN^–^ ratio. All FTIR spectra are normalized with respect to the center peak at ∼2072 cm^–1^.

The assignment of the CN stretch peaks of thiocyanate was further investigated by varying the overall SCN^–^ concentration at constant Li^+^ concentration in the HCE regime (i.e., [O]/[Li] < 5). To this end, different amounts of LiTFSI were added while keeping the Li^+^:G1:G2 ratio at 1:0.5:0.5 ([O]/[Li] = 2.5). The normalized FTIR spectra (Figure 3) show that increasing the amount of TFSI^–^ relative to SCN^–^, decreases the shoulder at ∼2043 cm^–1^ while increasing the shoulder at ∼2095 cm^–1^. This variation in the spectra with TFSI^–^ concentration not only indicates a change in the speciation of the thiocyanate but also shows that each shoulder band corresponds to a different ionic species.

**Figure 4:**
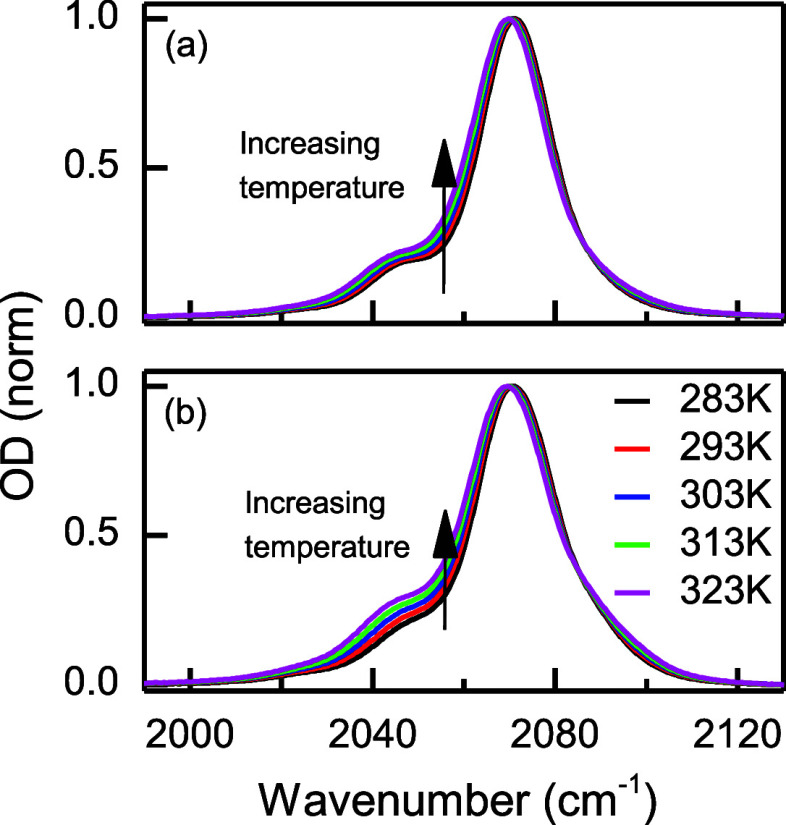
Temperature dependence FTIR. Panels (a) and (b) correspond to LiSCN-G4 and LiSCN-G1:G2, respectively, at temperature from 283 to 323 K.

Finally, temperature-dependent FTIR was also used to explore the equilibrium of ionic species in the G4 and G1:G2 SILs (i.e., [O]/Li = 5). The IR spectra as a function of temperature (Figure 4) show that both shoulders increase with temperature, regardless of the solvent. Again, this suggests that the SILs contain different ionic species, represented by the central peak and shoulders, which are in an equilibrium that is easily altered by changing the temperature. In addition, it is deduced that the three peaks correspond to related species since at higher temperatures the central peak transforms into the shoulders.

## Discussion

The Walden plot (Figure 1) shows higher ionicities for HCEs and SILs ([O]/Li ≤ 5) as compared to REs ([O]/Li > 5), regardless of the glyme nature and despite the higher viscosities of the HCEs and SILs. Previous studies have reported a similar decrease in ionicities when the [Li(G3)]TFSI and [Li(G4)]TFSI SILs were diluted with “noninteracting” solvents, such as hydrofluoroether, toluene, and diethyl carbonate.^56,93^ In the studied electrolytes, the SILs and HCEs have an increased number of charge carriers, but their molar conductivity steadily decreases due to a significant increase in their viscosity (see the Supporting Information). This decrease in ionic conductivity for HCEs and SILs is in agreement with a previous report.^46^ However, the ionicity in the highly viscous HCEs and SILs ([O]/Li ≤ 5) shows an increase, indicating a more efficient charge transport mechanism of the electrolytes.

Evidence of the change in the charge transport mechanism is also obtained from the Walden plot (Figure 1) of the REs ([O]/[Li] > 5) and HCEs/SILs ([O]/[Li] ≤ 5). In this case, a variation in the WP slope (Δ log Λ/ Δ log η^–1^) is readily seen when transitioning from one regime to the other. Note that the behavior of the LiSCN:glyme system is not that of the “ideal” KCl solutions since the latter shows a linear behavior in the Walden plot, consistent with a uniform conduction mechanism and the noninteractive nature of its ionic species.^94^ The G4 HCE/SIL regime ([O]/[Li] = 4–5) presents a lower WP slope (Figure 1) than G4 REs ([O]/[Li] > 5), which shows that the ionic conductivity has a larger change in the magnitude than the viscosity, or equivalently, G4 SILs are more efficient conductors than G4 REs. On the other hand, the slopes of RE and SIL for G1:G2 show a slight difference (Figure 1), with the latter appearing to be larger. This last result highlights the relatively small difference in the ionic conduction between the two regimes (RE and SIL) in the G1:G2 system. In contrast, the slope in the HCE regime of the G1:G2 electrolyte is lower than that of the G1:G2 REs and SILs but similar to that of the G4 SIL, indicating that the G1:G2 electrolyte becomes a more efficient conductor in the HCE regime and similar to that of the G4 SIL.

The effect of glyme identity on the transport properties was further evaluated by comparing the mixed (G1:G2) and pure (G4) glyme electrolytes at the same [O]/[Li] ratios. Both studied mixed and pure Li-glyme systems show similar transport properties in the RE regime of the electrolytes ([O]/[Li] > 5), as evidenced by their similar ionicities. However, in the SIL regime ([O]/[Li] ≤ 5), there is a clear difference between pure (G4) and mixed glymes (G1:G2). As shown by the ionicities (lower ΔW), a more efficient charge transport mechanism is observed for pure glyme in the SIL regime. The difference in ionicities between G4 and G1:G2 SILs is in agreement with the WP slopes, where the G4 HCEs and SILs present smaller slopes than the G1:G2 analogues (Figure 1). Again, this last observation further supports the presence of a more efficient charge transport in the pure glyme SIL compared to the mixed glyme SIL. Overall, the changes in the WP slopes confirm a change in the ion transport mechanism to one that is less influenced by the viscosity and more dependent on the salt concentration (i.e., [O]/[Li]) and chemical identity of the glyme.

The disparity in the relative change in the conductivity versus viscosity for SILs and HCEs is consistent with previous reports on SILs, where an ion transport mechanism less reliant on the vehicular mode of charge migration has been proposed.^40,48,62^ The proposed ion transport mechanism on highly concentrated electrolytes (∼3 M [Li(glyme)_*X*_]TFSI) is based on ligand- and/or anion-mediated ion hopping or ligand exchange.^34,39,40^ Therefore, for Li-glyme electrolytes at HCE and SIL regimes ([O]/[Li] ≤ 5), one can hypothesize that the Li^+^ coordination sites may not be fully occupied by the solvent, increasing the formation of bridged aggregates, which facilitates the making and breaking of ion pairs and, consequently, the ion transport. SIL studies have shown evidence of ∼3 and ∼20% free glymes in good and poor SILs, respectively. Thus, SILs have a significant population of Li^+^ not being fully solvated by the glyme molecules even when there are enough oxygen atoms in the solvent to fully coordinate all the cations.^95^ These previous experimental results revealed the existence of complexes in which multiple Li^+^ coordinates to the same glyme molecule and/or anion to fulfill its coordination. Moreover, a previous study showed that the presence of ionic clusters, aggregates, and/or CIPs can explain the large transfer number of a poorly conducting SIL (i.e., [Li(G3/4)TFA]).^96^ This suggests that such poorly conducting systems consist mainly of CIPs and exchangeable free glymes that facilitate Li^+^ transport.^59^ It is therefore expected that SCN^–^ should also undergo different ionic speciation. Indeed, the observed variations in the ionic transport properties of the studied Li-glyme electrolytes in the different regimes appear to be directly correlated to the ionic speciation observed in the FTIR spectra of SCN^–^ (Figure 2).

The ionic speciation of SCN^–^ in the FTIR spectra (Figure 2) shows at least three ionic species for the anion in the HCEs/SILs ([O]/Li ≤ 5), and one for the REs ([O]/Li > 5) regardless of the solvent. Most notably, the anion is found to be predominantly coordinated to Li^+^, forming CIPs, as deduced from the comparison with the TBASCN sample (Figure 2). For G4 and G1:G2, the REs show the same IR signatures, demonstrating the CIP as the anion speciation (Figure 2). For the HCE and SIL regimes, the SCN^–^ speciation is derived from the LiTFSI dilution experiment (Figure 3). In this case, it is observed that the ionic species corresponding to the high-frequency band (∼2095 cm^–1^) increases, while that of the low-frequency band (∼2043 cm^–1^) decreases when the concentration of SCN^–^ is decreased relative to the total Li^+^, which is kept constant through the addition of LiTFSI. Consequently, it is concluded that the high-frequency band is related to AGGs, having SCN^–^ coordinated with multiple Li^+^, i.e., [Li]:[SCN] > 1, while the low-frequency band relates to AGGs, with Li^+^ coordinated with multiple SCN^–^, i.e., [Li]:[SCN] < 1. In addition, the temperature-dependent FTIR experiment (Figure 4) agrees with the existence of related AGGs species and not simply CIPs, since the species corresponding to the central band convert to those of the shoulders at higher temperatures. In other words, the presence of only CIPs in the central band should lead to the formation of free anions at higher temperatures, which should appear only on the low-frequency side of the spectrum and at the same position as TBASCN. However, when the temperature is increased, a new band in the TBASCN location is not observed, and instead, a growth of the two shoulders is seen. Hence, these results provide experimental evidence for the formation of glyme/anion-bridged AGGs as previously hypothesized.^34,39,40^ In addition, it is shown that all of the AGG species are in equilibrium with interconversion energetics close to thermal energy (K_B_T).

**Figure 5:**
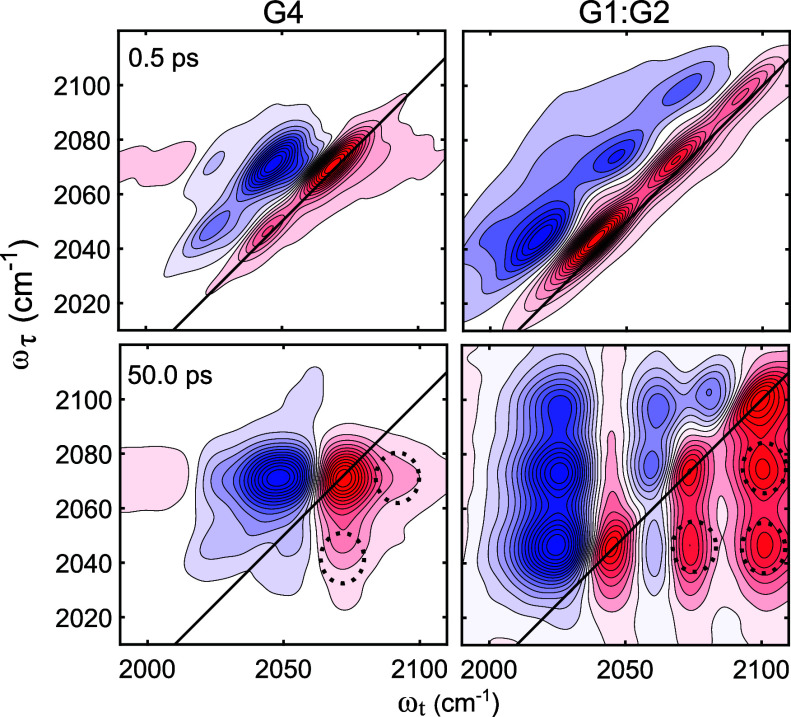
2DIR spectra in the CN stretch region of LiSCN in G4 SIL (left panels) and G1:G2 HCE (right panels) for *T*_w_ = 0.5 and 50.0 ps. The dashed circle shows the locations of the cross peaks.

The thermal equilibrium existing between the different AGG species in the Li-glyme electrolyte systems was demonstrated by 2DIR spectroscopy. The 2DIR spectra in the CN stretch region (∼2000 to 2150 cm^–1^) for the G4 SIL and G1:G2 HCE systems (Figure 5 and see the Supporting Information for the full *T*_w_ series) show not only the diagonal peak pairs (red and blue peaks) but also cross peaks (Figure 5 dashed circles) that grow with waiting time (*T*_w_). While the presence of the diagonal features has a direct correspondence to the FTIR spectra, the off-diagonal features correspond to anions that started at one frequency and ended at another during the waiting time.^97^ Therefore, the cross peaks could arise from either vibrational energy transfer or chemical exchange.^97^ However, the 2DIR spectra show the absence of cross peaks at initial waiting times and their growth with waiting time supporting a chemical exchange interpretation of the observed spectral features.^97−101^ The presence of chemical exchange between peaks confirms the thermal interconversion of the different AGG species present in the HCE and SIL regimes for both solvents (G4 and G1:G2) deduced from the FTIR. Overall, the IR experiments reveal the existence of multiple and thermally interconverting AGGs in the HCE and SIL regimes.

Previous studies utilizing electrophoretic NMR on [Li(G4)_*x*_]BF_4_ (*x* = 1, 2, 4, and 8) electrolytes have proposed the formation of charged clusters (asymmetric anionic and cationic) appearing at high salt concentrations.^93^ The observation of different AGGs in the SIL and HCE regimes, which are thermally interchanging, provides a molecular framework to explain the enhanced ionic transport of these systems compared to REs. It also explains the higher ionicity of SILs and HCEs containing G4, since these samples contain higher populations of AGGs than the corresponding G1:G2 SILs or HCEs, as evidenced by the size of their FTIR shoulder bands (Figure 2) and despite their lower salt concentration. Note that the ionicity of the SILs includes not only the ionic speciation in the system but also the charge transfer between ions.^102^ However, the investigated systems have the same chemical interactions besides the cooperativity effect of the larger glyme. Therefore, the changes in the ionicity observed upon formation of the SILs are likely caused by the change in ionic speciation. The results highlight the greater propensity of G4 to form ionic aggregates compared to the G1:G2 mixtures, which explains the more efficient ion transport observed for G4 SILs and HCEs.

**Scheme 2:**
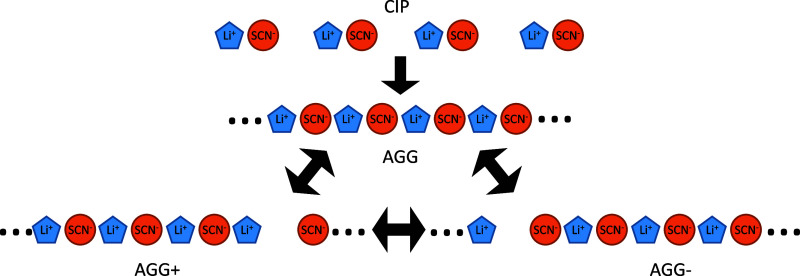
Cartoon Representation of the Proposed Chemical Equilibrium between the CIPs, Neutral Ionic Aggregates (AGG), and Charged Aggregate (AGG±) Species of LiSCN in Glymes

The following speciation model (Scheme 2) is proposed from all of the experimental observations and explains how the different CIPs or AGGs (charged and neutral) appear in Li-glyme electrolyte systems depending on the regime (concentration). In this molecular framework, the anion initially forms CIPs in the RE regimes ([O]/Li > 5) regardless of the solvent. The presence of CIPs explains the generally low ionicity of the REs^103^ since associated ions, such as CIPs, are less impacted by the electric field due to their neutral charge. In the HCE and SIL regimes ([O]/Li ≤ 5), the CIPs form neutral AGGs, which can dissociate to form charged AGGs (±). These latter species (AGG+ and AGG−) have a direct effect on the conductivity because they are not neutral, and thus they contribute to the ionic transport. Moreover, all these species undergo ultrafast thermal exchange as shown by 2DIR spectra (Figure 5) suggesting that making and breaking of aggregates play a key role in the efficient ionic transport mechanism observed for HCEs and SILs. The high population of charged AGGs in the G4 ([O]/Li = 4) and G1:G2 ([O]/Li ≤ 3.75) HCEs, as depicted in Figure 2, explains the enhanced ionicity of these systems. However, it is also evident that the charge transport is more efficient in pure glyme HCEs (G4) as compared to mixed glyme HCEs (G1:G2). For REs, the lack of AGG species reduces the propensity of ion pair making and breaking and, consequently, the conductivity. Therefore, the REs are dominated by low ionicities and vehicular ion transport, which are strongly influenced by viscosity.

**Figure 6:**
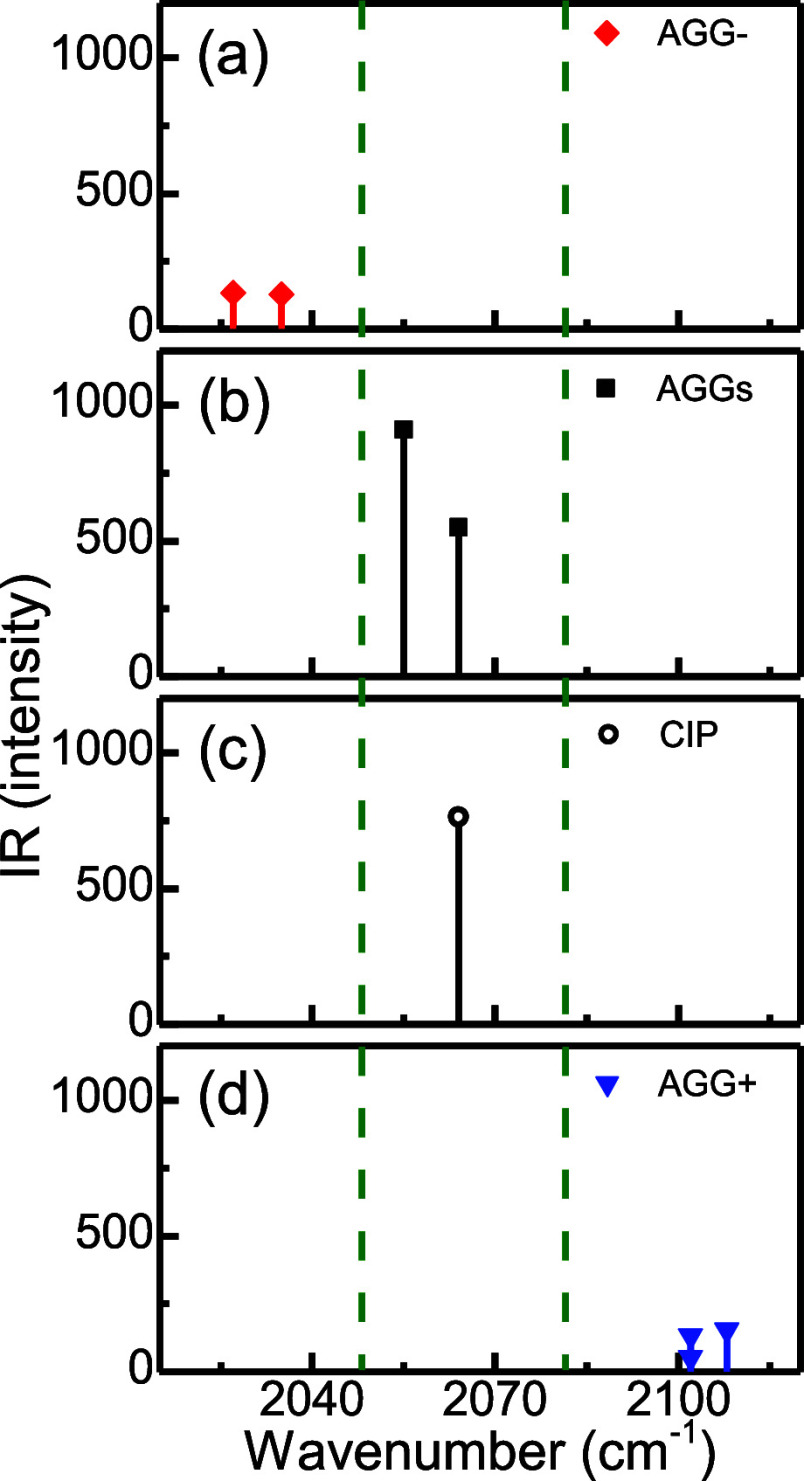
DFT calculated CN stretch IR frequencies of SCN anion in panels (a), (d) charged aggregates (AGG±), (b) neutral aggregates (AGGs), and (c) contact ion pair (CIP). The dashed olive line shows the three spectral regions.

Support for the proposed ionic speciation was obtained from DFT computations. To this end, the CN stretch modes were computed for the possible ionic species (see the Supporting Information), including CIPs, charged aggregates (AGG±), and neutral AGG. DFT calculations show three possible CN stretch frequency regions (Figure 6) in direct agreement with the experiments: low at ∼2030 cm^–1^, middle at ∼2060 cm^–1^, and high at ∼2100 cm^–1^_._ These IR signatures correspond to very specific species: the negatively charged AGGs (AGG−), CIPs and neutral AGGs, and the positively charged AGGs (AGG+), respectively. The computed IR signatures fully agree with the assignment of the bands to different ionic species derived from the experiment as AGG– contains more SCN^−^ and is located at low frequency, AGG+ contains less SCN^−^ and is located at high frequency, and neutral AGGs are positioned in between.

The ionic speciation as a function of the electrolyte regime allows us to rationalize the changes in the charge transport mechanism at the molecular level. It has been previously proposed that ion hopping is the conduction mechanism in HCEs composed of LiTFSI, with the anion coordinating and decoordinating from different Li^+^ centers.^104,105^ In this study, the ion pair making and breaking mechanism is deduced to be the dominant mode of charge transport for the Li-glyme systems in HCE and SIL regimes (i.e., [O]/[Li] ≤ 5).^100^ This mode of charge transport, which is partially decoupled from the electrolyte viscosity, explains the relatively high ionicities of the studied HCEs and SILs, regardless of the solvent identity and despite their significantly high viscosities. Previous studies have also proposed the existence of glyme-bridged aggregates, but this study probes and proves the presence of anion-bridged aggregates. Moreover, this study also reveals that the use of mixed glymes has a deleterious effect on the ionicity of the resulting HCEs and SILs. This detrimental effect is likely due to the fewer oxygen coordination sites in the shorter glymes, which reduces the propensity to forming glyme-bridged AGGs (both neutral and charged) and, consequently, the mechanism of charge transport via making and breaking of charged species. Therefore, the HCEs and SILs of Li-glyme electrolytes studied here show superior ionicity to REs, regardless of the chemical nature of Li^+^ solvation. The larger ionicity results in a more efficient charge transport mechanism involving charged species, AGG+ and AGG–, as opposed to the conventional vehicular mode relying on diffusion for REs. Note that the presence of aggregates not only produces a larger than expected ionicity in these SILs but also low conductive electrolytes.^59^ The latter is likely caused by the large dynamic ion correlations arising from the ionic aggregates as previously shown.^59^ In addition, the chemical nature of glyme solvation does not appear to have a significant effect on either the conductivity or the ionicity in the RE regime. Overall, the framework obtained by studying these systems provides a molecular description connecting the microscopic structure with the macroscopic properties of HCEs, SILs, and REs.

## Summary

This study shows that the LiSCN-glyme-based electrolytes (HCEs, SILs, and REs) are composed of coordinated ionic species in the form of CIPs and charged (AGG±) and neutral (AGG) aggregates. The HCEs and SILs ([O]/[Li] ≤ 5), despite having higher viscosities, exhibit enhanced ionicities when compared to REs. This change is attributed to the higher concentration of the charged aggregates in the HCEs and SILs, which facilitates viscosity-decoupled charge transport based on making and breaking of aggregates. The chemical nature of glyme solvation (G4 or G1:G2) appears to have a minimal effect on the transport properties of REs ([O]/[Li] > 5) due to the similarity in speciation of the REs regardless of the type of the glyme solvation. However, the use of mixed glyme solvation appears to have a detrimental effect on the ionicity of the HCEs and SILs due to a decreased propensity to form ionic aggregates in the shorter glymes, resulting in a charge transport that is more reliant on vehicular transport. This study expands our fundamental understanding of the relationships between the microscopic and macroscopic properties of glyme-based HCEs, SILs, and REs. Furthermore, the study suggests that the use of mixed glyme solvation as a tool for viscosity control may be more relevant for glyme-based REs ([O]/[Li] > 5) compared to HCEs or SILs ([O]/[Li] ≤ 5). Overall, the study not only provides a molecular framework for describing the solvation in glyme-based HCE, RE, and SIL-like electrolytes but also highlights the important role that the chemical nature of the glyme solvation plays in influencing their local structure as well as their macroscopic properties.

## Supporting Information Available

Complete 2DIR spectra waiting time series for 1:1: LiSCN in G4 and 1:0.5 LiSCN in G1G2; viscosity and molar conductivity as a function of fraction of lithium; solvation geometries of the calculated DFT structures; and the table of conversion from the LiSCN:glyme ratio to molarity to the [O]/[LI] ratio (PDF)
