# Supplementary material for: Transitioning from Regular Electrolytes to Solvate Ionic Liquids to High-Concentration Electrolytes: Changes in Transport Properties and Ionic Speciation
Source: J Phys Chem C Nanomater Interfaces. 2024 Jul 10;128(28):11522–33. doi: 10.1021/acs.jpcc.4c02248 (PMC11264273; doi:10.1021/acs.jpcc.4c02248)
Supplement: Supplementary file 1 — jp4c02248_si_001.pdf [file jp4c02248_si_001.pdf]

Supporting Information

Transitioning from Regular Electrolytes to Solvate Ionic Liquids to High Concentration Electrolytes: Changes in Transport Properties and Ionic Speciation

Ernest O. Nachaki† *and Daniel G. Kuroda*†*

†Department of Chemistry, Louisiana State University, Baton Rouge, Louisiana 70803, United States

*Address correspondence to dkuroda@lsu.edu

The complete 2DIR spectra

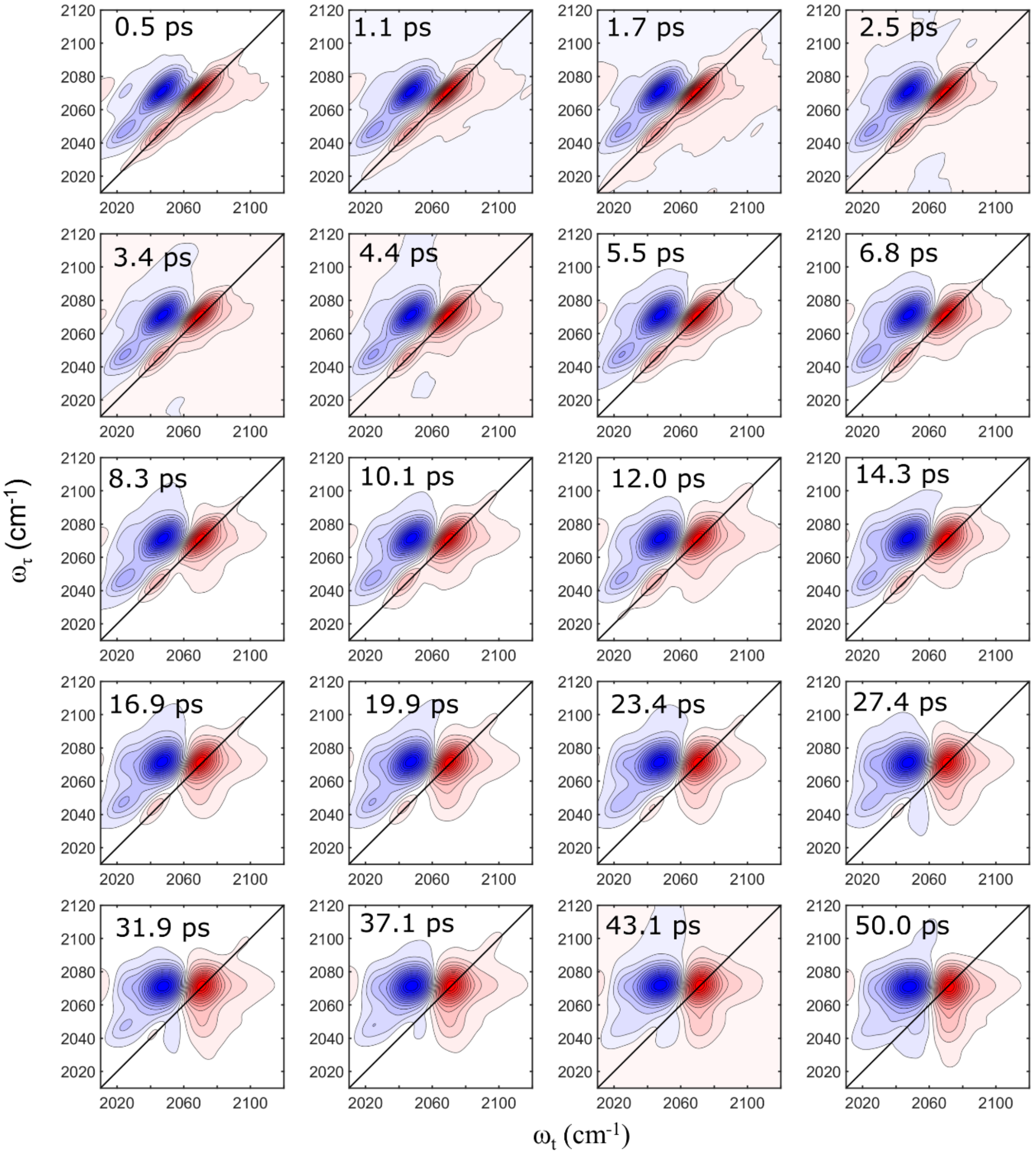

Figure S1. 2DIR spectra in the CN stretch region of 1:1 LiSCN in G4 with waiting times from 0.5 ps to 50 ps.

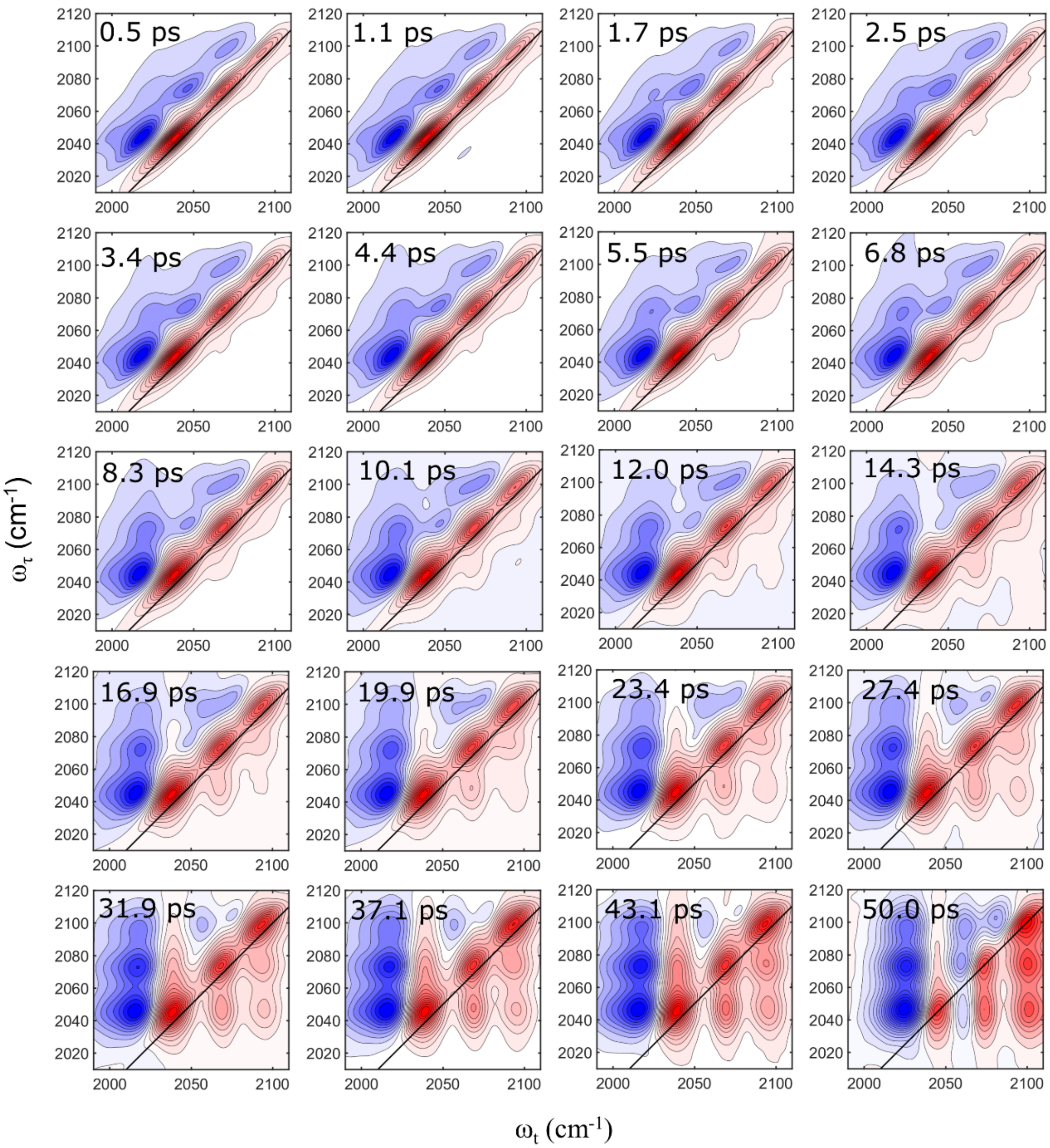

Figure S2. 2DIR spectra in the CN stretch region of 1:0.5 LiSCN in G1:G2 (1:1) with waiting times from 0.5 ps to 50 ps.

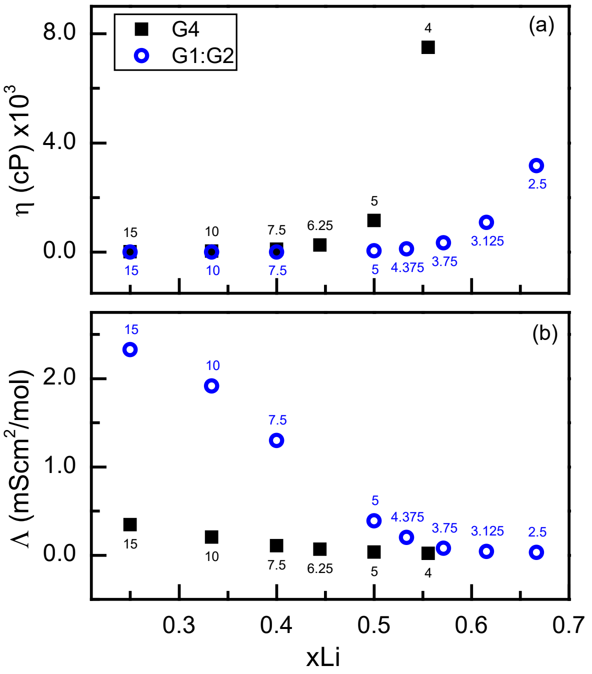

Figure S3. Transport Properties of LiSCN:Glyme as function of the fraction of Lithium and nature of glyme in the electrolyte, panel (a) Viscosity (η), and panel (b) the molar conductivity (Λ).

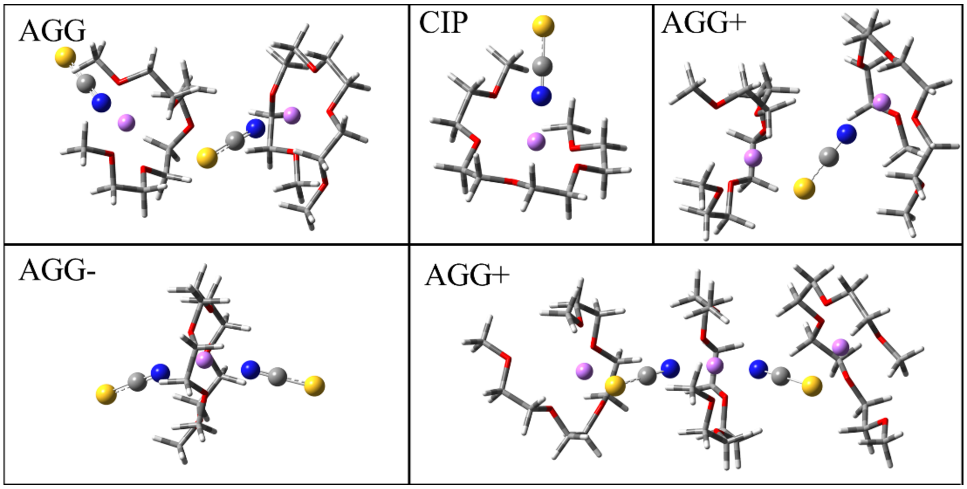

Scheme S1: DFT optimized solvation geometries of LiSCN in G4 involving neutral aggregate (AGG), negative aggregate (AGG-), CIP, and positive aggregates (AGG+).

Table S1. Conversion from LiSCN:Glyme ratio to molarity to the oxygen per Li^+ ratio [O]/[Li]. For G1:G2 mixture, 1:1 LiSCN:Glyme refers to LiSCN:G1:G2 = 1:1:1, to give a total of 5 oxygens of the glymes per Li^+.

LiSCN:G4	Molarity (M)	[O]/[Li]	LiSCN:G1G2	Molarity (M)	[O]/[Li]
1:0.8	5.67	4	1:0.5	6.23	2.5
1:1	4.54	5	1:0.625	5.35	3.125
1:1.25	3.64	6.25	1:0.75	4.58	3.75
1:1.5	3.03	7.5	1:0.875	3.99	4.375
1:2	2.27	10	1:1	3.59	5
1:3	1.51	15	1:1.5	2.48	7.5
			1:2	1.90	10
			1:3	1.29	15
